# Gastric and Typhoid Fever

**Published:** 1893-07

**Authors:** 


GASTRIC AND TYPHOID FEVER.—The following differential diagnosis has (*Med. Bull.*) lately been cited:

| Typhoid Fever. | Gastric Fever. |
|---|---|
| 1. It approaches slowly. | 1. It approaches, as a rule, rapidly. |
| 2. The temperature does not reach its highest point before the fourth day; never reaches 104 before the fourth day. | 2. The temperature reaches its highest point before the fourth and sometimes by the first or second day; often 104 on second day. |
| 3. Has bronchial coughs. | 3. Does not have a bronchial cough. |
| 4. Has a great tendency to bleed at the nose. | 4. Has very little of such tendency. |
| 5. Has tympanites. | 5. Does not have tympanites |
| 6. Has loose stools and diarrhœa. | 6. Bowels constipated, as a rule. |
| 7. Has rose-colored spots on the stomach. | 7. Never has rose-colored spots on the stomach. |
| 8. Has tenderness on pressure and gurgling in the right ileac region. | 8. Has not. |
| 9. Tongue characteristic. | 9. Has a tongue entirely different from typhoid fever. |
| 10. Has a duration of fever from 3 to 6 weeks; average 4 weeks. | 10. Has a duration from 3 days to 3 weeks; average 9 days. |
| 11. Does not originate *de novo*. | 11. Does originate *de novo*. |
| 12. Feebly contagious from person to person, and actively so from contaminated water. | 12. Not contagious. |
| 13. Cannot be arrested or aborted. | 13. Can very often be arrested or aborted. |
| 14. Temperature rises gradually from day to day. | 14. Temperature, as a rule, rises rapidly and suddenly. |

